# The anticipating brain is not a scientist: the free-energy principle from an ecological-enactive perspective

**DOI:** 10.1007/s11229-016-1239-1

**Published:** 2016-10-21

**Authors:** Jelle Bruineberg, Julian Kiverstein, Erik Rietveld

**Affiliations:** 1 Institute for Logic, Language and Computation (ILLC), University of Amsterdam, Oude Turfmarkt 141, 1012 GC Amsterdam, Netherlands; 2 Amsterdam Medical Centre, Department of Psychiatry, University of Amsterdam, 22660, 1100 DD Amsterdam, Netherlands; 3 AMC/Dept. of Philosophy/ILLC /Amsterdam Brain and Cognition, University of Amsterdam, Oude Turfmarkt 141, 1012 GC Amsterdam, Netherlands

**Keywords:** Free-energy principle, Predictive-coding, Skilled intentionality, Affordances, Enaction, Active inference, Action-readiness, Metastability

## Abstract

In this paper, we argue for a theoretical separation of the free-energy principle from Helmholtzian accounts of the predictive brain. The free-energy principle is a theoretical framework capturing the imperative for biological self-organization in information-theoretic terms. The free-energy principle has typically been connected with a Bayesian theory of predictive coding, and the latter is often taken to support a Helmholtzian theory of perception as unconscious inference. If our interpretation is right, however, a Helmholtzian view of perception is incompatible with Bayesian predictive coding under the free-energy principle. We argue that the free energy principle and the ecological and enactive approach to mind and life make for a much happier marriage of ideas. We make our argument based on three points. First we argue that the free energy principle applies to the whole animal–environment system, and not only to the brain. Second, we show that active inference, as understood by the free-energy principle, is incompatible with unconscious inference understood as analagous to scientific hypothesis-testing, the main tenet of a Helmholtzian view of perception. Third, we argue that the notion of inference at work in Bayesian predictive coding under the free-energy principle is too weak to support a Helmholtzian theory of perception. Taken together these points imply that the free energy principle is best understood in ecological and enactive terms set out in this paper.

## Introduction: finding a home for the free-energy principle

Anticipatory or predictive dynamics have been at the heart of a large number of influential and very diverse theories of mind, brain and skilled behavior more generally (Helmholtz 1860/1962; Gregory 1980; Grush 2004; Turvey 2010; Noë 2004; Stepp and Turvey 2010; Port and Van Gelder 1995; Thompson 2007; Chemero 2009). These approaches all share a focus on future-oriented activities, but they span a wide range of possible positions in the philosophy of mind. The apparent overlap disguises some fundamental theoretical differences and disagreements. It is within this incompatible plethora of broadly future-oriented frameworks for understanding cognition that one needs to situate the new kid on the philosophical block: the free-energy principle.

The free-energy principle (Friston and Stephan 2007) is a potentially unifying theory in theoretical neuroscience and theoretical biology, stating that all an organism needs to do in order to maintain its organization as an adaptive living system is to minimize its information-theoretic free-energy in its interactions with the environment. This minimization can be done by predicting or anticipating sensory input or by changing the environment to match what is anticipated. Adequate anticipation requires the organism to be tuned to its ecological niche in such a way that the coupled dynamics of the organism-environment system remain within a relatively small subset of states that maintain the organism’s viability in its econiche (Friston 2011). The promise of the free-energy principle is that it provides a theoretical framework able to unify the biological and the cognitive sciences. The free energy principle accomplishes this unifying work by showing how the organization and dynamics of living systems prefigures the organization and dynamics of cognitive systems. Living systems that regulate their interactions with the environment so as to maintain their viability, and cognitive systems that are the basis for complex capacities such as social cognition, cognitive control and language use, have fundamental organizational principles in common.^1^

The free-energy principle has often been combined with Bayesian predictive-coding,^2^ most notably in the work of its architect, Karl Friston (Friston and Stephan 2007; Friston and Kiebel 2009). The main idea of predictive-coding is that the brain generates top-down predictions that are matched bottom-up with sensory information, which results in prediction-errors.^3^ According to predictive-coding theorists, the brain is fundamentally in the business of minimizing prediction-errors (Hohwy 2013; Clark 2016). One of the aims of this paper is to better understand the relation between the free-energy principle and Bayesian predictive-coding. For the moment, it is important not to conflate them. The free-energy principle is a unifying framework for self-organizing living systems and is therefore not tied to any particular account of biological or neural functioning. Predictive-coding is a theory of neural functioning. How the latter relates to free-energy minimization is a question to which we will return later.^4^

Friston claims that the principle goes naturally together with older ideas about perception and cognition that date back to the nineteenth century psychologist Helmholtz. These ideas have been influential in more recent years in the work of the perceptual psychologist Gregory (1980) and the cognitive neuroscientist Frith (2007). Helmholtzians take perception to be “unconscious inference”, whereby the task of perception is to infer, based on top-down knowledge structures, the current causes of sensation. The process of inference is taken by them to work in ways that are analogous with scientific hypothesis-testing. On the other hand, the free-energy principle is consistent with many other accounts of self-organizing (living) systems such as synergetics (Tschacher and Haken 2007), global brain dynamics (Freeman 1987, 2000), metastability (Kelso 2012; Rabinovich et al. 2008), autopoiesis (Maturana and Varela 1980; but see Kirchhoff (2016)) and ecological psychology (Gibson 1979).^5^ Not all of this work is theoretically consistent with a Helmholtzian theory of perception, some of it is even explicitly aimed against such an account. *Our aim in this paper is to show that the free-energy principle and the Helmholtzian account of perception are conceptually independent.* One can explain how the free-energy principle applies to a biological system in terms of the dynamic coupling of the organism with its environment, we suggest. Whether this dynamic coupling is best described using the notion of “inference” we think is an open question. Within the Helmholtzian theory, the notion of inference is standardly understood as a probabilistic relation between prior beliefs, current evidence and posterior beliefs. However, the free-energy principle applies not just to humans but to all living systems, including the simplest of life forms such as bacteria. Friston employs the notion of “inference” even in these minimal cases of simple life forms. This is clearly stretching the meaning of “inference” beyond its normal usage. We question whether the activities of simple life forms such as *E. Coli* can be taken to be inference-involving. In previous work, we have argued for an “ecological-enactive” understanding of the free-energy principle under the umbrella of the Skilled Intentionality Framework (SIF) (Bruineberg and Rietveld 2014, figure 1; Rietveld et al. forthcoming; see Ramstead et al. (2016); Kirchhoff (2015) and Gallagher and Bower (2014) for similar arguments). One aim of this paper is to build on our earlier work in part by situating our ideas in relation to those of Hohwy, Friston and Clark, in order to highlight some key differences. We think that Friston’s important theoretical work has been mistakenly aligned with Helmholtzian ideas about perception (Hohwy 2013; Clark 2013; Friston and Stephan 2007). The stress on Helmholtz leads to important aspects of the free-energy framework being missed in the philosophical discussion. More specifically, action and perception are not understood in the context of self-organization, and we will argue that this leads to philosophical errors.

Our argument proceeds as follows. In the first part of the paper, we introduce the free-energy principle in more detail. We emphasize the biological motivation for the free-energy principle (as found in Friston and Stephan 2007 and Friston 2011), because it shows that first and foremost the free-energy principle does not apply to brains or epistemic agents, but to embodied living systems as a whole. Second, the biological motivation for free-energy minimization highlights the continuity of mind and life (Thompson 2007), and hence the overlap with the enactive approach.

In Sect. 2, we introduce Bayesian predictive-coding as a theory of brain function and show how it has been taken to form a natural partner with the free-energy principle. We argue against the dominant Helmholtzian interpretation of Bayesian predictive coding in part on the grounds that it provides what we demonstrate to be a problematic conception of the brain as working in much the same way as a hypothesis-testing scientist. In Sect. 3 we then show how Friston’s concept of probabilistic inference, central in much of his work on the free-energy principle, can be given a deflationary interpretation using concepts from dynamical systems theory. We further argue that the free energy principle and Bayesian predictive-coding constrained by the free-energy principle, contrary to conventional wisdom, provides no support for a Helmholtzian inferential theory of perception.

## The free-energy principle and its enactive foundations

One of the most fascinating questions in the biological sciences is how living systems can produce and maintain their organization in the face of a dynamic environment. A second, equally important and fascinating, question is how biological processes can give rise to minds. The continuity of life and mind thesis, as defended by enactivist philosophers of biology (Jonas 1966/2001; Di Paolo 2005; Thompson 2007), states that both these questions should be tackled at once. Life and mind share the same basic underlying principles. It is exactly this organisational continuity of life and mind that is the starting point of Friston’s free-energy principle, suggesting common ground between the free-energy principle and enactive philosophy of biology. Friston agrees that the defining feature of living systems is the way in which “biological systems [...] maintain their states and form in the face of a constantly changing environment” (Friston 2011, p. 92). Self-maintenance and self-production are the defining features of autopoietic (self-producing) systems, suggesting that there might be an intimate relation between free-energy minimization and autopoiesis as a defining characteristic of life [but see Kirchhoff (2016)].

From this conception of life, one can derive a theoretical framework for thinking about perception and action.^6^ Free energy is a function of the organism’s sensory states and the organism’s internal dynamics (called a generative model).^7^ Roughly, free energy is a measure for the ever present dis-attunement between environmental dynamics and internal dynamics (Bruineberg and Rietveld 2014). Free-energy can be minimized on short time scales by making the environment conform to the internal dynamics (“action”) or by making the internal dynamics conform to the environmental dynamics (“perception”). This proposal by Friston is, admittedly, a rather unorthodox view of perception. Sensory states here are the proximal stimulation of the organism which can only be changed by acting on the world. Rather than talking about perceptual states we (Bruineberg and Rietveld 2014; Kiverstein and Rietveld 2015) prefer to speak of states of action-readiness. States of action-readiness are the internal states of the individual that, given its sensory states and abilities, prepare the animal to achieve a grip on a particular situation.^8^

The free-energy principle describes how the very same properties that define life are also essential for cognition, understood as the capacity to regulate interactions with the environment. As Clark has suggested, the free-energy principle may “reveal the very deepest of links between life and mind, confirming and extending the perspective known as “enactivist” cognitive science” (Clark 2013, p. 24).

Friston formalizes the self-maintenance aspect of autopoiesis in terms of *Shannon information*: of all possible states the organism could find itself in, the organism must find the right subset of states that allow its organization to persist in its energetic exchanges with the environment. The claim is that there is a probability distribution over all possible states the organism can find itself in. At any point in time, this distribution is sharply peaked around certain values specifying conditions of the organism that are necessary for its viability and survival or—more exactly—that define the characteristic phenotypic state of the organism. For example, human body temperature has a high probability of being around 37 $^\circ$C and a low probability elsewhere. Mathematically, this means that the probability distribution of the variable body temperature has low *Shannon* *entropy* and that the event ‘measuring a body temperature of 37 $^\circ$C has low *surprisal*,^9^ while measuring a body temperature of 10 $^\circ$C has a very high *surprisal *(for homeothermic organisms like ourselves). Importantly, whether a temperature has low or high surprisal is relative to different species of animal. Birds have a different average body temperature than humans, while ectothermic animals such as lizards have a different distribution altogether.

What holds for internal variables holds just as well for places in the environment. For whales, being in deep sea is an event with low *surprisal*, and being on shore has high *surprisal*, while this is reversed for humans. Hence, the particular embodiment or biological organization of an animal and the environmental conditions of the animal necessary for its viability constrain each other.^10^ This is an illustration of the mutualism of animal and environment, taken together they form a complementary pair:

We commonly talk of the organism and the environment and of the adaptation of one to the other ...as if there were first an organism and an environment and then some adjustment of one to the other; but when we come to an analysis of the factors involved, it is quite necessary to start from the unity of function and see that the distinction of organism and environment arises because of adaptation in that process, not vice versa (Dewey 1976, p. 275, as quoted by Costall 2004, see also Maturana and Varela 1980).

So far, all we have said is that the embodiment of the animal implies a range of environmental conditions in which it is able to prosper and that surprisal is a measure that captures this relationship. As such, the free energy principle is just an information-theoretic formalization of the observation that living systems are homeostatic (Bernard 1865/1927) or homeodynamic (Yates 2008) systems. What is required is an account of *how* living systems are able to find the right subset of states so as to maintain themselves within viable bounds.^11^ The ideal temperature of a human is determined by its embodiment: at 37$^\circ$ the enzymes regulating our metabolism perform optimally, while the metabolic cost of maintaining body temperature is still manageable within certain environmental conditions.

The challenge that the homeostatic system faces is that of regulating the animal’s interactions with the environment so as to keep the animal within this viable regime, without “knowing” about the animal’s own viability conditions. Surprisal is relative to this unknown probability distribution, thus an organism has no means of evaluating surprisal directly. Since surprisal is a more general term in information-theory, we will call this special case of surprisal, only related to the embodiment of the agent, *embodied suprisal* or *surprisal* $_{E}$. We will call the unknown distribution, which formally describes the conditions of viability of the animal, the *bodily distribution*. How then does the organism succeed in finding the right subset of states?

This is where the free-energy principle may hold a solution. The main insight of the free-energy principle is that information-theoretic free-energy is always greater than (or equal to) surprisal^12^ $_{\mathrm{E}}$ and is thus an upper bound on surprisal$_{\mathrm{E}}$. By minimizing free-energy, the organism will implicitly minimize surprisal$_{\mathrm{E}}$ as well, and hence remain within viable bounds. Free-energy is accessible to the organism because it is a function of two quantities (i.e., probability distributions) that the organism embodies: a generative model and a variational density that is entailed by a system’s internal state. Mathematically, the generative model is thought of as the probability of the co-occurrence of a sensory state and a state of the environment. The variational density is a proxy for the real bodily (environmental) probability distribution (e.g. the distribution of temperature). Typically, one assumes that this distribution is Gaussian; making it fully specifiable by only its mean and standard deviation. Changing the variational density (i.e. changing its mean or standard deviation) will give different values for the free-energy. What is needed then for free-energy *minimization* is for the organism to be able to change the variational density or to change the environment in a way that reduces free-energy. Friston appeals to the derivative of free-energy with respect to internal states (that encode the variational density) and to sensory states.^13^ This implies nothing more, we take it, than that the organism regulates its interactions with the environment and its own internal conditions based on what it takes to be the norm of maintaining its own viability or flourishing more generally.

We argued so far that surprisal$_{\mathrm{E}}$ (for instance related to temperature) cannot be evaluated directly because the ‘true’^14^ embodied probability distribution is unknown. Instead, based on the generative model the system itself generates a variational density about its own optimal temperature that can be used to evaluate “how far off” the current temperature is. We will use the general information-theoretic notion of surprisal to describe this discrepancy. Free energy is always greater than the average surprisal$_{\mathrm{E}}$. This can be seen intuitively: if the internal mechanism regulates temperature to be 38$^\circ$, while our embodiment requires 37$^\circ$, there will be a big discrepancy (‘surprisal$_{\mathrm{E}}$’) over time. The closer the variational density approximates the bodily distribution, the lower the surprisal$_{\mathrm{E}}$ will be. Hence minimizing the discrepancy between the ‘predicted’ and the ‘actual’ temperature requires that the following condition be met: the internally generated (variational) distribution and the actual distribution must be mutually compatible with respect to the sensory constraints (in the sense of being sufficiently similar enough in their dynamics).^15^

The organism changes the variational density by changing its internal dynamics with respect to the constraints of the environment. Friston calls this “perceptual inference”. The latter is not sufficient for minimizing surprisal$_{\mathrm{E}}$ (and therefore survival, if the logic of the free-energy principle holds). In order to minimize surprisal$_{\mathrm{E}}$ an organism needs to change its sensory states, which it does through action. Perception, understood in Friston’s terms as changing the internal dynamics of the organism, will only lower the upper-bound on surprisal$_{\mathrm{E}}$ (i.e. free-energy) and not change surprisal$_{\mathrm{E}}$ itself. Crucially, to change *surprisal* $_{\mathrm{E}}$ itself, the organism needs to change its sensory states *by acting* on its environment. Suppose a human being finds itself with a temperature of 39.8$^\circ$. If it would be accommodating enough to change its internal dynamics so that this was its expected temperature, it would not survive for long. What matters for the organism is avoiding finding itself in such a state by changing its sensory states through acting in the world (e.g. by going to the doctor).

**Fig. 1:**
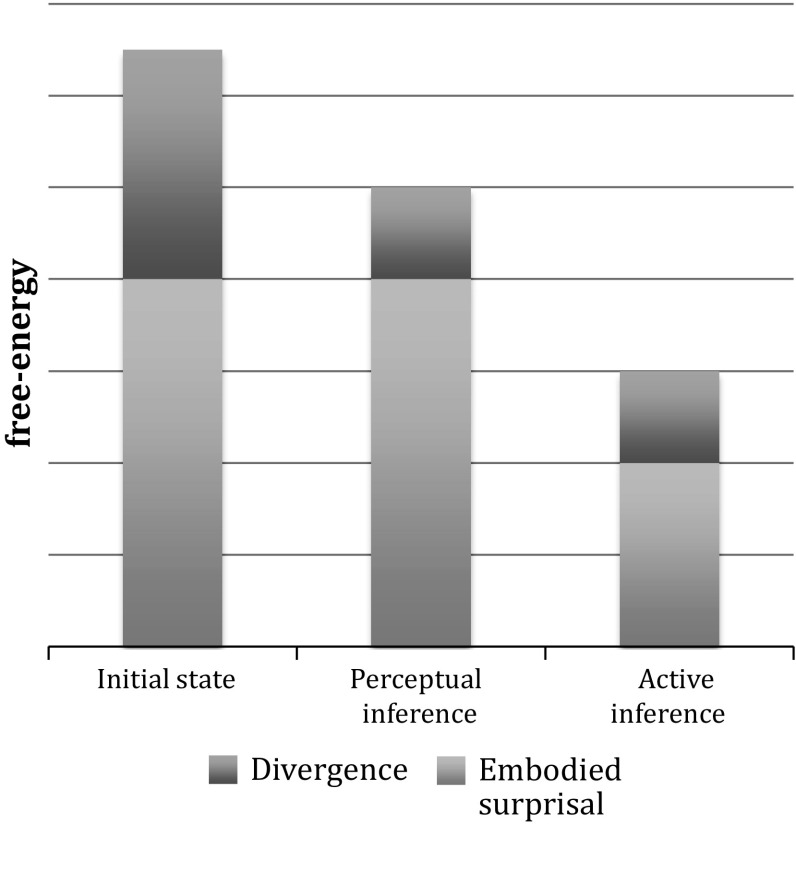
Schematic depiction of how free-energy is composed of divergence and embodied surprisal (suprisal$_{\mathrm{E}}$) and how these components change with perceptual and active inference respectively (see text). Perceptual inference can only reduce divergence, but not embodied surprisal. Only active inference can reduce embodied surprisal

Action is therefore necessary for minimizing surprisal$_{\mathrm{E}}$; this cannot be done on the basis of perception alone since perceptual inference cannot reduce surprisal$_{\mathrm{E}}$. Mathematically, this can be seen by the fact that one can write the free-energy term as a Kullbeck–Leibler^16^ divergence between the variational density and the posterior plus the term for surprisal$_{\mathrm{E}}$. Minimizing this term by only changing the internal states (perception) minimizes the KL-divergence, and not the surprisal itself (see Fig. 1, see also for instance Friston 2011, Figure 1). We will in the following refer to KL-divergence as “divergence”.

So within the free-energy framework, it is *action* that does the work of actually minimizing surprisal. Actions change an organism’s relation to the environment, thereby changing the sensory states of the organism, a process that Friston calls active inference (2012). Free energy, as we understand it (Bruineberg and Rietveld 2014), is a measure of the dis-attunement of the internal dynamics and the environmental dynamics: it is low when the sensory states are anticipated, by the animal, and high when they are not. The free energy principle says that minimizing free-energy is a necessary^17^ condition for living systems to maintain their organization in their econiche. What is crucial for the organism, is that it anticipates the kind of interactions with the environment that contribute to its flourishing (such as for example eating food, staying warm, avoiding a passing car or learning to read). The function of the generative model is therefore not to provide the agent with a representation of the dynamical structure of the environment per se. It mediates the organism’s interactions with its environment in such a way that a *robust *brain-body-environment system is maintained. The generative model is not a model of the environment as it happens to be. It embodies in its organization and structure longer-term regularities between action, environment and the state of the organism. How can we understand the relationship between the niche and the generative model?

Friston claims that “an agent does not *have *a model of its world, [but rather] it *is* a model” (Friston 2013b). One thing this means is that the free-energy principle does not just apply to the brain, but rather to the organization of the biological agent as a whole in its econiche. Friston himself states, somewhat provocatively, that: “each [...] agent embodies an optimal model of its econiche” (Friston 2011). In previous work (Bruineberg and Rietveld 2014) we have provided a minimal yet still practically useful interpretation of “*being* a model of its econiche”. Minimally this implies: (1) that the agent embodies in its structure and organization skills that complement the econiche and (2) that the coupled dynamics of organism and niche^18^ together lead towards some dynamical equilibrium or grip on the situation. The free-energy principle provides an explanatory framework for understanding the coupling, or attunement, between internal and external dynamics. Following Friston, we are led to the conclusion that, because of the organism’s skills and affordance-related states of action readiness, the organism itself *is* its own best model of the causal structure of its niche, a paradoxical sounding twist on the familiar embodied theme that “the world is its own best model” (Brooks 1991).

The main motivation for the free-energy principle is the biological requirement for self-organization and self-maintenance. The causal processes that hold internal variables and environmental variables within bounds are coupled. For instance, a decrease in blood sugar level of a mammal might alter the anticipated sensory input such that it anticipates finding a food source, hence making the behaviour of the animal conform to its metabolic needs. Auletta (2013) provides a good example of how a coupled informational (sensorimotor) and metabolic system can provide a model for bacterial chemotaxis.^19^ The system that is minimizing free-energy is nothing less than the whole self-organising and self-regulating affordance responsive system.

The free-energy principle then reveals the deep continuity between mind and life that Clark (2013) has hinted at as being reminiscent of enactive approaches towards life and mind^20^ (Varela et al. 1991; Thompson 2007; Stewart et al. 2010; Colombetti 2013; Di Paolo et al. 2010). From our enactive point of view, it is the myriad of self-maintaining and self-producing processes that make up the animal, that give the animal a lived perspective on its environment. Metabolic and thermal disequilibria structure the way in which the world is perceived in the sense that it makes the individual organism selectively open and responsive to *relevant* affordances, to the relevant possibilities for action the environment offers. An apple might invite eating when I am hungry, and a blanket might solicit wrapping around me when I am cold. What counts as minimizing surprisal is thus relative to the current state and situation of the animal and its lived perspective on the many affordances offered by its environment. In acting in order to minimize surprisal, the individual is responsive to only the disequilibria with the environment that are relevant given its current state and situation.

In many kinds of animals, these disequilibria might be experienced as an affective tension or something-to-be-improved. The animal is drawn to act by the relevant affordances in its situation, so as to reduce this tension, which results in its improving its grip on its environment. We call this the tendency towards an optimal grip on the situation, which we take to be a basic concern of every living animal (Rietveld 2008a; Bruineberg and Rietveld 2014; Kiverstein and Rietveld 2015). This basic concern to improve grip is a precondition for and structures the animal’s lived perspective on the basis of which certain affordances offered by the environment stand out as significant or relevant. Minimizing surprisal then can be understood as an animal reducing its disattunement with the environment. In doing this, the animal improves its grip on the environment.

There are two points we therefore wish to stress that will be important for our later argument. First, surprisal is relative to an animal with an affective perspective on its ecological niche. The individual cares about something, namely about improving or at least maintaining grip on its changing situation. Friston emphasizes self-maintenance as a distinguishing feature of living systems from other self-organizing systems, where self-maintenance is understood as the ability to change their relationship with the environment and maintain thermodynamic homeostasis (Friston and Stephan 2007, p. 5). We have characterized this ability as an instance of what in philosophical phenomenology is described as being moved so as to tend towards an optimal grip. Thus on our view self-maintenance implies an affective perspective on its environment. Certain aspects of the animal’s econiche stand out as significant for the organism on the basis of its basic concern to improve its grip on the environment, and to reduce its disequilibria with the environment.

Second, we have argued that free-energy minimization is best understood in terms of the disattunement of the internal and external dynamics of the animal and its environment. It is the whole organism in regulating its interactions with the environmental landscape of affordances that is minimizing surprisal. We will see in the next section, how Jakob Hohwy argues that minimizing surprisal is the evolutionary function of the brain. Hohwy takes the minimization of surprisal to be a function of the brain in the same sense as the function of the heart is to pump blood. We’ve argued by contrast that minimization of surprisal is done by the whole organism being drawn to act on relevant affordances in ways that result in the reduction of dis-attunement with the environment.

## The Helmholtzian brain as a crooked scientist

Jakob Hohwy has drawn on the free-energy principle to develop a theory of the predictive brain that is very different from the ecological and enactive one we have just proposed. He interprets the free-energy principle using older ideas from Helmholtz (1860/1962) and Gregory (1980), following up on suggestions that can also be found in Friston’s writings (see for instance Friston and Kiebel 2009). Hohwy rightly points out that the Helmholtzian theory of perception is strongly internalist in a way that challenges enactive and ecological approaches towards the mind. In *The Predictive Mind* (2013), Hohwy has given one of the most comprehensive and careful expositions of the prediction-error minimization framework (PEM) to date. In a more recent follow-up paper, he has the following to say about what his interpretation of PEM implies for a philosophical account of the mind-world relationship:

PEM should make us resist conceptions of this relation on which the mind is in some fundamental way open or porous to the world, or on which it is in some strong sense embodied, extended or enactive. Instead, PEM reveals the mind to be inferentially secluded from the world, it seems to be more neurocentrically skull-bound than embodied or extended, and action itself is more an inferential process on sensory input than an enactive coupling with the environment. (Hohwy 2014, p. 1)

Generally in formulations of PEM, brain processes are taken to work according to a unifying principle related to but not identical with the free energy principle. PEM claims this unifying principle is the minimization of prediction-error between the actual inflow of sensory input occurring over multiple temporal and spatial scales, and the flow of sensory input the brain predicts based on its top-down knowledge. Sensory processing in the brain is not a bottom-up process of feature detection but instead consists of the top-down prediction of sensory signals based on hierarchically organised models of environmental causal regularities. What gets conveyed through bottom-up processing are weighted prediction errors that arise when there is a discrepancy, or “prediction-error” between incoming inputs, and what the brain expects. Sensory processing thus becomes about predicting sensory input. By minimizing prediction-errors over time (given a suitable generative model) the system “will come to infer the hidden state of the environment”.

Hohwy equates PEM with the view of perception as knowledge-driven perceptual inference as advocated by Helmholtz and Gregory. The logic is the following: the brain only has access to its own predictions and to prediction-errors and based on these it has to infer the hypothesis that best explains away current prediction error. Perception then essentially is hypothesis-testing in which the brain seeks to explain away prediction-errors by finding the hypothesis that best explains the available sensory evidence. The ‘unconscious inferences’ that lie between a sensation and a perception are much like the inferences that scientists draw (Gregory 1980; Hatfield 2009). It is the inferred hypothesis about the causes of sensory input that provides our epistemic access to the external world. The brain only has access to the ways its own patterns of neural activity and spike trains flow and alter. The true states of the external environment are hidden from the brain beyond the veil of sensory information. Any epistemic access to the world is therefore, according to Helmholtzians like Hohwy, indirect based on the hypothesis that best explains away current prediction error.

This Helmholtzian line of reasoning assumes however a distinction between sensation and perception that is problematic for ecological and enactive cognitive scientists. The general disagreement between inferential and ecological approaches to perception concerns the richness or sparsity of our sensory contact with the environment. For the Helmholtzian, sensations are impoverished and need to be enriched by top-down knowledge before the perceiver can know what is out there in the world. This view of perception as starting from impoverished and ambiguous sensations does not fit with the richness of the ecological context. For the ecological theorist, the perceptual system is attuned to directly (i.e. non-inferentially) pick up on environmental regularities in the organism’s niche. Within that framework, perception is understood in terms of lower-level phenomena like resonance and attunement, mediated by the organism’s skills.^21^

As we have argued for above, the animal species and the ecological niche are co-specifying. The niche of the animal is best understood as a landscape of affordances that reflects the perceptual capacities and other abilities of a kind of animal belonging to a particular form of life (Rietveld and Kiverstein 2014). Affordances are more precisely defined as relations between aspects of the material environment and abilities available in a ‘form of life’ (ibid., p. 337).^22^

The lived perspective of an individual organism reflects its needs, concerns and abilities and determines which affordances are relevant in a particular situation. Hohwy is absolutely correct to pick up on a tension between Helmholtz and conceptions of the mind like our own that stress openness to the world. However, unlike Hohwy we take the free-energy principle to challenge those that would follow Helmholtz.

Hohwy conflates PEM with the free-energy principle. He writes: “Since the sum of prediction error over time is also known as free-energy, PEM is also known as the free-energy principle (Friston and Stephan 2007).” (Hohwy 2014, p. 2) We believe this is confusing, if not simply wrong. There is a difference between the free-energy *bound* and the free-energy *principle*. The free-energy bound is the computational trick of reducing an (intractable) integration problem to an optimization problem by introducing a variational density.^23^ The free-energy *principle*, on the other hand, is tied to the minimization of surprisal and therefore (by the logic of the free-energy principle) to self-organization and what we call the tendency towards an optimal grip on affordances. As we have shown in Sect. 1, one important consequence of the free-energy principle is that it is *only action that minimizes surprisal* $_{E}$. Perception, understood as changing the internal dynamics of the organism, will only lower the upper-bound on surprisal$_{E}$ and not change surprisal itself.

For Hohwy, perceptual inference is changing the hypothesis so that it fits the data, whereas active inference is changing the data so as to fit the hypothesis. Both are different strategies for minimizing prediction-error. Hohwy has a particular way of adding action to perceptual inference.^24^ In perception the brain tests its current hypothesis of the world by predicting the sensory input or data it would expect on the assumption that its current hypothesis is true. It then compares the predicted data with the actual data and goes on to adapt its hypothesis to improve the fit with the actual data. The brain laboriously changes and fine-tunes its hypothesis as experimental results come in. These iterations of hypothesis-testing are what Hohwy has in mind when he talks about *perceptual inference*. He introduces active inference by appealing further to the analogy between perception and scientific hypothesis testing (Hohwy 2013, p. 77). Scientific hypothesis testing is not just passively recording results of an experiment, but carefully setting up experiments and actively intervening in the chains of causes and effects in order to disentangle the causal web. In action the brain controls movement, sampling the environment with the aim of matching actual sensory input with predicted sensory input. According to this framework, the role of action is to support perceptual inference and makes predictions more reliable (Hohwy 2013, p. 79).

However, we have seen that form the perspective of the free-energy principle, *perceptual inference without active inference does not make sense*, since only action minimizes surprisal. Let us return to the example of temperature we used in Sect. 1. If you find yourself too hot, one way to reduce your temperature is by taking a cold shower. *Action* here is doing the work of reducing surprisal$_{\mathrm{E}}$, because of its effect on the animal’s relation to the environment, taking it closer to an optimal bodily condition. Perceptual inference also works in the service of actively improving the organism’s grip on the environment. We’ve followed Friston in characterising this form of inference in terms of change in the animal’s internal (endogenous) dynamics. Perceptual inference can thus be thought of as patterns of action readiness. In the case of the shower, the sensation of being too hot shapes your perspective on the environment in such a way that the cold shower now stands out as inviting or attractive (whereas normally it probably would not). Perceptual and active inference are not two distinct strategies for minimizing prediction-error (each with their own so-called ‘directions of fit’), as Hohwy suggests. Perceptual and active inference are entangled parts of a single process of readying the organism to act in such a way as to improve its grip on the environment. Perception, understood as changing the organism’s internal dynamics reflects the organism’s pattern of action readiness and thus its selective openness to affordances. The organism is ready to act on relevant affordances so as to improve its grip on the environment thereby reducing surprisal. There is no separation between perception and action because perception on our account just is the organism’s preparing itself to act in ways that reduce surprisal, thereby improving grip on its environment both by getting ready for what is to come and by engaging with affordances offered by it in action. The environmental affordances scaffold the individual’s actions. Action does not have a world-to-mind direction of fit, as Hohwy argues, because in acting the agent has no intention or goal in mind. Instead the agent is drawn to act based on its disattunement with the environment. It is the affordances relevant for reducing this disattunement that drive the actions of the agent.

Both Hohwy and Friston acknowledge the central role for action in the minimization of surprisal (Hohwy 2013, pp. 84–88), but we think they fail to realize the damage it does to their Helmholtzian commitments. As mentioned earlier in this paper, on a Helmholtzian account of the mind, the aim of perceptual inference can be said to be to infer the most likely cause of sensory input, the more objective the better. However, we would argue that a perfect hypothesis that precisely represents the state of the environment is worthless if it does not specify what action minimizes surprisal, or improves grip. Hohwy recognizes this point when he writes as follows: “perceptual inference can make you perceive that you are hurtling towards the bottom of the sea with something heavy around your feet but cannot do anything to change that disturbing sensory input which is fast taking you outside your expected states.” (Hohwy 2013, p. 85) It is only by action that you can do something about this disturbing sensory input by, for instance, untying the heavy object from your feet. However, active inference of the type involved in such a life or death situation, would be ecologically useless if it did not predict adaptive actions that would improve my situation in this life threatening environment. The possibility of cutting the rope with my Swiss army knife in my pocket should become the relevant affordance that drives my actions now.

The upshot of this is that my brain is not, and should not behave like an exemplary scientist. If my brain really is a scientist, then it is heavily invested in ensuring the truth of a particular theory, which is the theory that “I am alive”. This is a fundamental prior belief that drives all action; namely, I exist and I will gather all the evidence at hand to prove it. It will only make predictions whose confirmation is line with this hypothesis. It does not give competing hypotheses a fair chance and is extremely biased in the way it interprets the data. It decides on the outcome of an experiment beforehand (my staying alive) and manipulates the experiment until the desired result is reached. If my brain is a scientist, it is a crooked and fraudulent scientist—but the only sort of scientist that can survive an inconstant and capricious world.^25^ The hypotheses that the brain in reality is biased in favor of, are hypotheses that predict the animal will tend towards grip on environmental affordances.

The interesting question in active inference is therefore not, as Hohwy claims, how the brain can use available sensory input to accurately reconstruct the hidden state of affairs in the world (Hohwy 2014, p. 1). The interesting question is rather how the space of possible ‘hypotheses’ is always already constrained in such a way as to make the animal improve its grip on the environment.

Hohwy might seem to recognize this point when he discusses the limitations of actions understood as active sampling of sensory input. However, he attempts to offer an account of PEM that abstracts away from questions about how the space of hypothesis is already constrained: “I will not engage these types of questions directly here. I am primarily interested in what the account says about our understanding of the world and our place in it as perceivers and agents.” (Hohwy 2013, p. 88) We, however, think that this is where the free-energy principle starts to get interesting: one cannot understand our place as agents in the world without taking into account self-organization. The free-energy principle with its focus on self-organizing dynamical systems is fundamental to answering these questions about the mind-world relation. The Helmholtzian metaphor of the brain as hypothesis-testing scientist is ill-equipped to capture active inference under the free-energy principle for the simple reason that it triggers the wrong questions. The central question for the organism is not what the state of affairs of the environment is (although this might be relevant for achieving certain forms of grip), but rather which actions will minimize surprisal, where surprisal is taken relative to an encultured, skilled and embodied agent. The organism is always acting on the basis of a basic concern to improve grip, and this biases and informs all of its actions. This basic concern is taken for granted or presupposed by the Helmholtzian.

This presupposition shows up for instance in the way in which active inference is informed by and depends upon the goals and intentions of the agent. For example, suppose I am falling down the stairs. I could perfectly predict myself tripping down the stairs and breaking my neck. Navigating the stairs is seen by the Helmholtzian as the goal of the agent, and relative to that goal active inference works so as to bring about perceptions that take you closer to accomplishing that goal. However, it remains a mystery why the agent has selected the goals that it has in this situation. Implicit in this appeal to goals and intentions is the basic concern of the organism towards improving its grip on its environment. The point per se is not about prediction-error minimization, because my brain can be correctly minimizing prediction-error as I fall down the stairs. Although my sensations might tell me it is very likely that I am tripping down the stairs, my brain *needs* to treat this as a highly unlikely event and do as much as possible to return to a more likely situation (i.e. balancing on two feet). These considerations are echoed in Friston’s (2011) account of embodied inference:

I model myself as embodied in my environment and harvest sensory evidence for that model. If I am what I model, then confirmatory evidence will be available. If I am not, then I will experience things that are incompatible with my (hypothetical) existence. And, after a short period, will cease to exist in my present form (Ibid., p. 117)

The Helmholtzian is thus already taking for granted without explaining something which is central to our account, the lived perspective and the concerns of the organism. Rather than simply presupposing goals and intentions, we suggest that the basic concern of the organism is tending towards improving its grip on available affordances in its environment.

Unlike Hohwy (2014) and Clark (2013)^26^ we follow Friston in taking the free-energy principle to be the biological principle of living systems as a whole, and not only of the brains of living systems. Goals and intentions ought to not be something external to the free energy principle, but rather should be explained by the principle itself. The Helmholtzian account does not in itself provide any explanation of the goals of the organism, and why the organism selects the goals it does. It rather specifies prediction-error minimization in relation to those presupposed goals without offering any explanation of the process by which the goals themselves are formed. By contrast our account understands free-energy minimization in terms of the tendency towards an optimal grip on available affordances. It thereby shows how selective openness to relevant affordances follows from acting according to the free-energy principle. There is therefore no need to presuppose goals and intentions whose origins remain quite mysterious, or for an additional account of goal and intention formation.

Our concept of the tendency towards an optimal grip additionally puts the Bayesian notions of precision and uncertainty in a different perspective.^27^ The traditional function of uncertainty in Bayesian accounts of perception is to modulate the impact of sensory perturbations (‘prediction-errors’) on the internal dynamics (‘hypothesis’). If the agent has high confidence in its sensory input and low confidence in its current hypothesis, then the sensory prediction-error will greatly shift the hypothesis. In the reversed case (low sensory confidence and high internal confidence), the probability density will be unaltered.

Confidence however cannot be a function of sensory evidence alone taken in isolation from contexts of skilled action and engagement with the world. The central question for the agent and thus for active inference is not to settle on which hypothesis is true, but on *what needs to be done*. Even as all sensory evidence points to me standing under a shower that is too hot, I *need* to treat this as an unlikely event and arrange my *actions* accordingly. Moreover, most of our actions are extremely context-sensitive: the ringing phone solicits answering when I am alone, for example, but not when I am having a conversation. Situations typically offer multiple possibilities for action, and the degree to which each of these possibilities stands out as salient or relevant is due to precision-modulation.^28^ Crucially, such precision-modulation is structured by an agent’s skills and concern—their acquaintance with a normative socio-cultural practice (e.g. when it’s acceptable to answer your phone); their habits (getting a cup of coffee before starting work); their bodily needs (eating an apple when hungry). While engaged in a conversation a buzzing phone leaves us cold (does not alter internal dynamics, has low precision), while in another context it solicits answering (high precision, impacts internal dynamics). Precision-modulation based on these kinds of factors, shapes the salience and relevance of the field of affordances with which agents engage. We cannot understand our place as agents in the world without taking into account the wider contexts and situations in which skilled action takes place.

## Inference behind a Markov blanket

Both Hohwy (2014) and Clark (2013) take the brain to occupy an epistemic position that implies a Helmholtzian theory of perception. They borrow from Eliasmith the notion of a perspective, to be able to specify for each neuron or brain mechanism what information it has access to in a strict sense:

A ‘perspective’, as I shall use the term, is a relation between an information processor and a transmitter of information. Perspective is determined by *what* information is available to an information processor from a transmitter (Eliasmith 2005, p. 99, italics in original).

The notion of information implied here is just information in terms of (meaningless) energy transfer and should not be confused with intentional or semantic content. As Eliasmith puts it, the ‘perspective’ is a simple relation between a transmitter and a receiver of information. Assigning such perspectives is a useful tool for tracking information flows and covariance relations in complex systems like the brain. Eliasmith goes on to make the trivial sounding claim that “[A]n animal (and each of its information processing sub-components) can only access information available through sensory receptors” (ibid., p. 100). It is now a short step to the conclusion that since all the brain has direct access to are the changes that are taking place in its sensory registers, the only route to knowledge of what it is in the environment that is causing its changing sensory input must be through inference of some kind. Clark captures this type of reasoning in the following passage. We quote him in full since it is precisely this line of reasoning that we wish to challenge in this final part of our paper. Clark writes:

For, the task of the brain, when viewed from a certain distance, can seem impossible: it must discover information about the likely causes of impinging signals without any form of direct access to their source. Thus, consider a black box taking inputs from a complex external world. The box has input and output channels along which signals flow. But all that it “knows”, in any direct sense, are the ways its own states (e.g., spike trains) flow and alter. In that (restricted) sense, all the system has direct access to is its own states. The world itself is thus off-limits (though the box can, importantly, issue motor commands and await developments). The brain is one such black box. How, simply on the basis of patterns of changes in its own internal states, is it to alter and adapt its responses so as to tune itself to act as a useful node (one that merits its relatively huge metabolic expense) for the origination of adaptive responses? Notice how different this conception is to ones in which the problem is posed as one of establishing a mapping relation between environmental and inner states. The task is not to find such a mapping but to infer the nature of the signal source (the world) from just the varying input signal itself. (Clark 2013, p. 3)

Clark seems to imply in this passage that there is a strong epistemic boundary separating the brain from the outside world. The brain has to infer the hidden state of the world that lies beyond the veil of sensory information. Any epistemic access to the world has to be inferential, because the brain has no direct access to the causes of its sensory information. Based on the flow and alteration of its spike trains, the brain needs to internally reconstruct the causal structure of the world outside. This would seem to challenge the openness of the organism to the environment, suggesting instead that the organism is secluded and cut-off from the environment.^29^

**Fig. 2:**
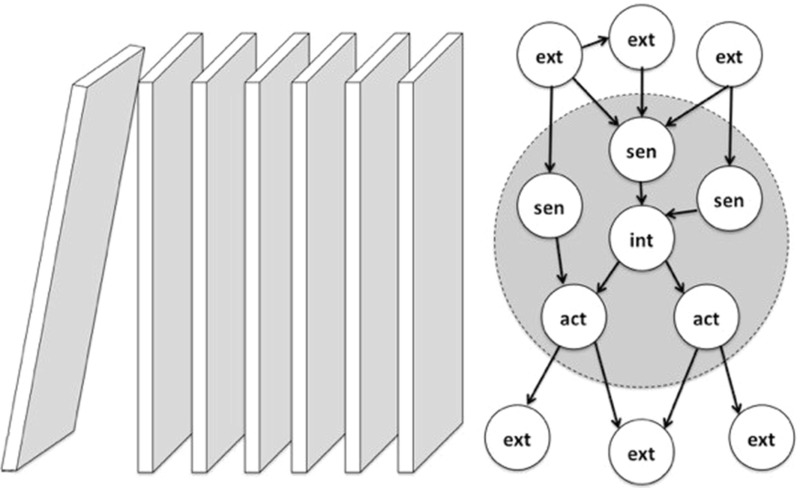
*Left* A domino chain reaction as an exemplificiation of a Markov process. Every domino is only dependent on its previous domino, just as, in the figure on the *right*, every node is only dependent on its neighboring nodes. *Right* A schematic depiction of a Markov blanket, a spatial generalization of a Markov process. The *gray circle* represents the Markov blanket of a node, consisting of internal state (int), its children (the action states, act), its parents (sen), and parents of children (sen), with parent/child being understood in terms of cause/effect

We will argue that the boundary between the organism and the environment is real, but it is not the kind of boundary that hides causal structure in the environment from the organism.^30^ We make this argument from simulations of very minimal cases of life and cognition as discussed by Friston (2013a). In this paper, Friston shows two things. First, that coupled ergodic dynamical systems with a Markov blanket are a sufficient condition for free-energy minimization and second, that such systems will spontaneously occur in a simulation of a “primordial soup” with relatively arbitrary assumptions.^31^ The Markov assumption, as it is known from statistics, is a conditional independence of a node with its predecessors, given its immediate predecessors (or ‘parents’). For instance, in a domino chain reaction, the behavior of a given tile is only dependent on the behavior of its predecessor in the chain. Although the behavior of the predecessor is dependent on the rest of the causal chain, given the behavior of its predecessor, the domino of interest and the rest of the causal chain are statistically independent (Fig. 2).

The Markov blanket is an extension of the Markov assumption in the sense that it makes a node or a subgraph conditionally independent from the rest of the network given its neighboring nodes or ‘Markov blanket’ (Pearl 1988). Markov blankets are mainly used in the context of Bayesian graphical models, but as Friston (2013a) shows, they are all pervasive in natural systems dominated by short-range interactions. Due to the Markov blanket, Friston argues, a random dynamical system can be described naturally in terms of ‘internal’ and ‘external’ states ‘shielded off’ by a blanket of ‘perception’ and ‘action states’. This allows for an inferential understanding of coupled dynamical systems. Friston claims that, over time, the intrinsic dynamics of the internal states (something like electrochemical dynamics) will start to ‘infer’ the causally disconnected states of the intrinsic dynamics of the environment (that are ‘hidden’ behind the Markov blanket).

Markov blankets introduce what Eliasmith calls a perspective of an animal on its environment. Do Markov blankets imply the inferential seclusion of the animal from its environment? We think this does not follow at all, and rather points to a direct coupling between animal and environment. Friston’s simulation of the primordial soup shows that what he calls ‘inference’ is a natural consequence of processes governed by Newtonian and electrochemical interactions, completely understandable in terms of the basic and natural phenomenon of self-organization that Friston describes using the tools of graph theory. The notion of “inference” Friston is using is much more minimal than the one typically employed in philosophy, and refers to the mutual information (mutual predictability). Inference, for Friston, is the process that leads to nodes inside of a Markov blanket and nodes outside of the blanket having high mutual information. We understand inference as implying a probabilistic relation between representational states. However, in the primordial soup simulation, we doubt that it makes sense to ascribe representational states to the systems that are being modeled. Instead, we can explain the process that leads to mutual information more parimoniously in terms of synchronization.

The simulations in Friston (2013a) show that any dynamical system A coupled with another B can be said to “infer” the “hidden cause” of its “input” (the dynamics of B) when it reliably covaries with the dynamics of B and it is robust to the noise inherent in the coupling (Bruineberg and Rietveld 2014, p. 7). We question whether it makes sense to call this inference, given that the process that leads to high mutual information need not be thought of as representational. Friston recognizes that the internal and external dynamics of an animal in its environment are coupled through something called generalized synchrony. Generalized synchrony is a well-known phenomenon in physics, stemming from Huygens (1673) study on the synchronization of clocks. It is worth taking a closer look at this phenomenon very briefly because it is the minimal basis for his notion of inference as these concepts are understood by Friston in his writings on the free-energy principle.^32^

**Fig. 3:**
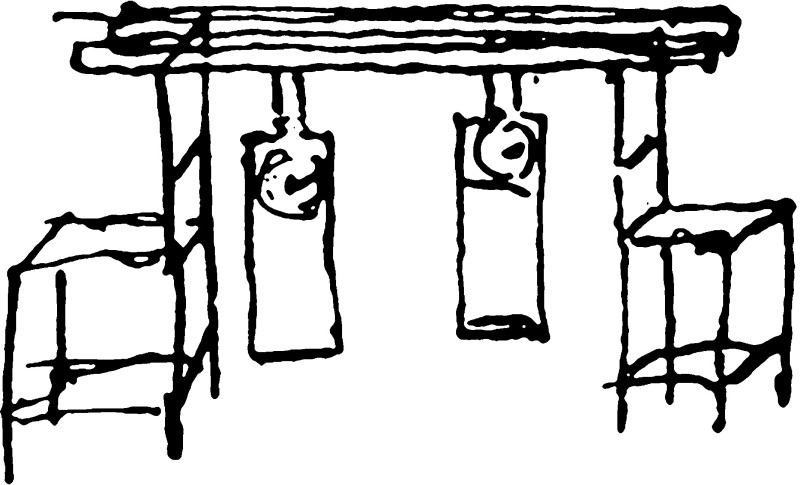
Original drawing from Huygens’ C. Horoloqium Oscilatorium. Two oscillating clocks hanging on a suspension beam that is itself able to move

Huygens observed that two pendulum clocks in the same housing start to synchronize through the tiny movement of the beam on which they are suspended, or the table on which they are placed. Such synchronization is a general and common phenomenon in non-linear dynamics (Strogatz 1994). When different time-scales are involved, one often speaks of ‘enslavement’, while when both systems exhibit similar typical times-scales one speaks of ‘entrainment’. One can understand these dynamics in terms of a synergetic ‘circular causality’ (Tschacher and Haken 2007). In systems with ‘circular causality’, there is no clear difference between internal dynamics attuning to external dynamics and vice versa (or clock 1 attuning to clock 2 and vice versa), rather both systems are bidirectionally coupled and reduce the disattunement between them until equilibrium (synchronization) is reached.

In this example the suspension beam, or table, acts as a Markov blanket. That is to say, the two clocks are statistically independent conditioned upon the movement of the beam. This is clear from the fact that no synchronization occurs when the beam is held fixed.^33^ When, for instance, the intrinsic dynamics of the two clocks are too different, no synchronization will occur.

Based on Friston (2013a), one would be required to give an inferential interpretation of the coupling of the two clocks, in which one clock “infers” the state of the other clock hidden behind the veil of the connecting beam. In this example, each clock is a ‘generative model’ of the dynamics of the environment (the other clock), coupled through the Markov blanket of the connecting beam. The synchronizing clocks are therefore excellent examples of *being a model* (discussed in the previous section). Clock A is a model of clock B if clock A resonates with clock B. Whether synchronization (or “inference”) occurs or not, is dependent on properties that have to do with the whole system: the period of the two clocks should not be too dissimilar; the beam should not be too rigid and not too flexible, and should be flexible in the right direction etc. We think such an inferential interpretation is unnecessary: there is no special inferential system inside of the Markov blanket, but the synchronization *is* the process of achieving high mutual information. This is emergent from the critically balanced coupled dynamics of the whole system.

Our point here is that, at least at this minimal level, there is no privileged explanatory role for the system that is defined ‘within’ the Markov blanket in achieving high mutual information with states of the environment. In the case of the two clocks, there is a perfect symmetry between ‘internal’ and ‘external’ dynamics. In the ‘primordial soup’ example of Friston (2013a), both the ‘organism’ and the ‘environment’ consist of essentially similar subsystems with electrochemical internal dynamics. It is only when these subsystems are similar enough that they will appear to exhibit active inference (i.e. to synchronize). Again, what counts as similar enough is dependent on the system as a whole.^34^ Given that attunement or synchronization takes place and given the ‘environment’, an observer can make predictions about the structure of the ‘agent’. In line with the free-energy principle, one can show that the average information-theoretic surprisal of the coupling is minimized when the dynamics of coupled systems are synchronized. In this sense, synchronization is a form of free-energy minimization, which can be given a Bayesian interpretation (the posterior divergence goes to 0 when the clocks synchronize). By synchronizing, clock 1 can be interpreted as maximising its Bayesian model evidence for the state of the environment. Although one can describe the behaviour of these systems in terms of probabilistic inference, this is unnecessary. The process of achieving high mutual information can better be understood in terms of the coupled dynamics of the system as a whole.

One might object that there is still a non-trivial boundary separating the system from its environment in both the examples discussed above. Both systems are causally interacting through a Markov blanket. We agree, but we do not think this has any implications that conflict with the arguments of this paper. The importance of such a boundary for living organisms has been central in the autopoietic approach from the very start (Maturana and Varela 1980; Varela et al. 1991). The mathematical methods used in ecological psychology also rely heavily on synergetics, synchronization and entrainment (Stepp and Turvey 2010; Stepp et al. 2011; Haken et al. 1985), all presupposing *coupled* (rather than fused) dynamical systems. If this is the only kind of boundary that stems from the free-energy principle, then there seems to be nothing in the idea of probabilistic inference per se that challenges enactive cognitive science (Bruineberg and Rietveld 2014; Clark 2015a). In other words, *if* the role played by the concept of inference in FEP is no more cognitively demanding than generalized synchrony, *then* it provides no grounds for distinguishing radical enactive approaches to cognition from those that are based on FEP.^35^

One might object that there is an important difference between simple life-forms or primordial soup simulations and more complex systems that predictive-coding is seeking to model. The behavior of the first class of systems might be understood in terms of dynamic coupling and reducing disattunement. The second class of systems necessitates the explicit computation of prediction-error based on a generative model and requires a more epistemic understanding of the internal workings of the animal: wherever there are systems that can select actions so as to deal with the absent or lie in the future, this requires explicit representational knowledge structures about these distal affairs, one might think (Seth 2013; Clark, personal communication).

However, as Orlandi (2015) points out as well, it is not clear that the structure of the generative model should be thought of as representational. We follow ecological psychology in arguing that the environment is rich with information that the perceptual system is able to directly access, because of our evolutionary history and the abilities and skills we learn. People and other animals (for instance primates) are situated in a very rich landscape of affordances (Rietveld and Kiverstein 2014). We proposed that the generative model is best thought of as a dynamical system of (affordance related) states of action readiness that reflect^36^ the hierarchical and temporal organization of the changing environment. As the animal develops skills, the generative model becomes more and more sensitive to the relevant particularities of the situation, and opens the animal up to the relevant affordances available in the environment.^37^

Some of these abilities and skills for engaging with the world may be reused in ways that may explain our capacities for planning, imagining and reasoning about counterfactuals (Anderson 2014). Other abilities for planning and imagining might come from the ways in which we actively structure the environment to scaffold our thinking (Rietveld and Kiverstein 2014). The exact balance of environmental and agential components is an open empirical question to be settled on a case-by-case basis (see the ethnographic description of architects at work in Rietveld and Brouwers (2016)). However, we believe that the richness of the landscape of affordances in which human embodied minds are situated will be an important part of the answer.

We wish to make three final points before we conclude. First, the explicit computation of a prediction-error will be based on structures that represent and encode long term information about causal regularities in the environment. We have argued that for many cases of free-energy minmization, it is unnecessary to make appeal to this type of structures within the organism. We leave it as an open question when, and if at all, it is necessary to explain the internal structure of the organism by appeal to representations. For our purposes we prefer to think in terms of affordance-related states of action readiness also for understanding forms of what is traditionally called ‘higher’ cognition. Second, the free-energy principle provides a clear functional continuity between simple living systems and complex ones like humans. Predictive-coding theories understood in abstraction from the free-energy principle run the risk of missing this important functional continuity, understood by us in terms of the tendency towards an optimal grip. Third, one of the big questions for future work in neuroscience is how more locally mechanistic accounts of brain functioning, like predictive-coding, can be integrated with a more broadly complex systems perspective on the brain. Phenomena like entrainment and synchronization seem to play a crucial role in brain functioning (Varela et al. 2001; Engel et al. 2001). We think the “ecological-enactive” reading of the free-energy principle provides a necessary framework for carrying out this integrative work. This is something one would fail to accomplish if one would exclusively focus on predictive-coding and ignore the free-energy principle.

## Conclusion

The Helmholtzian interpretation of the anticipating brain brings with it a problematic conceptualization of the boundary separating the organism from the environment. The environment becomes a hidden cause that must be inferred from ambiguous sensory input, which is all the brain has access to. However we’ve argued above that there is no reason to accept such a Helmholtzian interpretation of the relation between the organism and its environment. Moreover, such an interpretation is in deep tension with the free energy principle according to which the brain is a part of a larger coupled system that on the basis of its coupling is constantly reducing disattunement with the environment. Through the organism’s minimization of free-energy, the brain’s internal dynamics are normally adequately attuned to the external dynamics of the environment.

Central to the predictive processing framework is the notion of a generative model. On the Helmholtzian interpretation, a generative model is a model that captures the causal structure of the agent’s environment. The generative model is then used to generate ‘mock’ sensory input that is then compared to actual sensory input to compute prediction-errors. Such an interpretation introduces a functional asymmetry between (top-down) predictions and (bottom-up) prediction-errors. Top-down predictions are produced on the basis of internally realized knowledge structures (Clark 2016). This may raise the worry that our ecological-enactive interpretation fails to accommodate this functional asymmetry.^38^ Without these internally realized knowledge structures, we just have complex looping interactions between brain, body and environment that mysteriously reduces disattunement.

On our interpretation, the function of the generative model is not to provide the agent with a representation of the dynamical structure of the environment per se, but rather to steer its interactions (over multiple timescales) with its environment in such a way that a *robust *brain-body-environment system is maintained. The organism’s internal structure and organization is then understood as multiple simultaneous and coupled affordance-related states of action-readiness that together shape (through top-down precision-modulation) the salience of solicitations in the environment. The self-organization of these states of action-readiness allows the animal to tend towards an optimal grip on the multiple relevant affordances in the situation. There is an asymmetry between the sensory inputs that flow from the environment and the anticipations of sensory input based on the organism’s history of interactions with the environment. However, we have been arguing that the internal dynamics can be understood in terms of patterns of action-readiness that constitute selective openness to multiple affordances simultaneously rather than from internalized knowledge structures. As we have proposed in earlier work (Bruineberg and Rietveld), the tendency towards an optimal grip on multiple affordances simultaneously can be explained as a metastable attunement to environmental dynamics. This metastable attunement allows for rapid and flexible switching between relevant action possibilities (Kelso 2012; Rietveld 2008b; Bruineberg and Rietveld 2014). Crucially, once we dispense with misleading/distracting talk of probabilistic inference, it is no longer necessary to understand past experience and learning as encoded in the form of representational knowledge structures. Instead we understand past learning and experience as manifest in the skilled animal’s anticipatory dynamics to act in ways that improve grip on the affordances on offer in the situation.

Grip, as so conceived, is certainly not “the new representation” as Andy Clark has suggested (Clark 2015a). On Clark’s view, the generative model is estimating the current state of the organism in its environment, something that is only possible because of the knowledge the agent has of the world. On our view of the generative model, it is preparing the agent to perform actions that improve grip on multiple simultaneously relevant affordances in the situation. This is something that is only possible because of metastable attunement to environmental dynamics that make the agent open and ready for dealing effectively and flexibly with what the future may offer.

## Footnotes

1 Whether the free-energy principle is able to live up to these theoretical promises is also dependent on its interpretation. We think that an enactive understanding of the free-energy principle, stressing mind-life continuity and self-organization will solve some conceptual problems, which we highlight later.

2 Also called prediction-error minimization (PEM) by Hohwy (2013, 2014) and predictive processing by Clark (2013, 2015a, 2016).

3 For excellent introductions to predictive-coding see (Clark 2013; Rao and Ballard 1998; Friston and Stephan 2007).

4 It is important to distinguish between the information-theoretic *quantity* of free-energy and the free-energy *principle*. Predictive-coding is always formally related to the former, but not necessarily to the latter.

5 Self-organization is a central notion in Friston’s work. All these references can also be found in for instance, Friston (2000, 2012, 2013a) and Friston et al. (2012).

6 Taking this starting point is nothing new. A notable precursor in this way of thinking is physicist Erwin Schrödinger. In *What is Life?* (1944) Schrödinger starts from general thermodynamic considerations and develops a theory of metabolism. In an important sense, we take the Free Energy Principle to be an information-theoretic extension of Schrödinger’s work.

7 The concept of the generative model here should be understood in a particular way as a system of multiple interacting states of action–readiness (see, Bruineberg and Rietveld 2014). We expand on this point below.

8 We expand on this point on page 13 of the manuscript.

9 Shannon entropy is the expected or average surprisal and surprisal is related to an event with a particular outcome.

10 In forms of life with sociocultural practices this environment is highly complex and best understood as a sociomaterial environment (Mol 2002). The FEP is not limited to understanding adaptation to an environment offering resources for meeting basic biological needs such as food, but can also be applied to adaptation to the practices found in the human social environment. The landscape of affordances in the human form of life is very rich (Rietveld and Kiverstein 2014; Bruineberg and Rietveld 2014, figure 1).

11 In this section, we focus on biological examples to highlight the link between homeostasis, self-organization and the free-energy principle. Ultimately, the free-energy principle should help us to understand all improvements an individual makes in a situation. We will come back to this in the last section.

12 Formally, free-energy is defined as surprisal$_{\mathrm{E}}$ plus the KL-divergence between the recognition density and the embodied distribution. Since, KL-divergence is strictly positive, free-energy is an upper bound on surprisal$_{\mathrm{E}}$.

13 We explain what Friston means by an internal state later in this paper when we introduce the notion of a Markov blanket.

14 The ‘true’ distrubution is a technical term in statistics. The relevant distinction is that between a ‘true’ and an ‘empirical’ distribution’. In this context, the ‘empirical’ distribution is the variational density that estimates the ‘true’ embodied distribution.

15 Of course this assumes that the generative model is sufficiently in accordance with the bodily distribution. This requires an evolutionary and developmental explanation that takes into account the variability of the animal’s niche or the human form of life. In itself, the notion of free-energy minimization is applicable also on phylogenetic and ontogenetic timescales (Friston 2011, Figure 10).

16 The Kullbeck–Leibler divergence is the “extra” surprisal obtained for approximating the *bodily distribution* with a generative model. Intuitively, when throwing a fair dice, there is an expected surprisal per throw. When falsely expecting the dice to be skewed, there is on average an extra amount of surprisal gained. The difference between the two is the KL-divergence.

17 Free-energy minimization is sufficient for self-maintenance in the sense that it guarantees the system to frequent an invariant set of states. However, this is not sufficient for a demarcation of living from non-living systems (since a simple homeostat meets these criteria). We believe additional (enaction based) conditions are needed (see for instance Froese and Stewart 2010). We thank reviewer 2 for pressing us on this point.

18 Econiche, here and elsewhere in the paper, is understood as the landscape of available affordances to a certain animal species or form of life, see figure 1 in Bruineberg and Rietveld (2014).

19 In fact, Auletta (2013) mentions the genetic system as a third subsystem that constrains both the metabolic and the sensorimotor system over long time scales. Since we only deal with perception and action in this paper, and not with development and evolution, the genetic system should not concern us here.

20 See quote on page 5.

21 As Orlandi (2015) points out, there is an interesting historic continuity between Gestalt psychology, ecological psychology, connectionism and predictive-coding, wheras there is a discontinuity between predictive-coding accounts and a Helmholtzian theory of perception. We think that this continuity between ecological psychology and predictive-coding is important and becomes undeniable in the context of the free-energy principle.

22 Note that the human form of life includes socio-cultural practices and many of the affordances of the human (sociomaterial) environment and the corresponding abilities have a place within in these practices.

23 This method is used extensively in machine learning under the heading of variational inference or variational Bayes (for an introduction, see Murphy 2012, Chapters 21 and 22). This technique is mainly used in forms of inference where one wants to investigate which causal structure most likely generated the data.

24 As originally conceived, active inference (Friston et al. 2011) subsumes both perception and action.  It purports to generalize the Helmholtzian perspective to include action, rendering them inseparable.  We will return to this later.

25 Thanks to Karl Friston for suggesting this formulation.

26 Since his 2013 paper, Clark seems to have changed considerably his position regarding the relation between embodied cognition, Helmholtzian perception and predictive-coding. In recent work he (Clark 2015a), is no longer committed to an inferential Helmholtzian view of perception, but rather talks about grip. However, also in this work, it remains unclear where goals and intentions come from.

27 We thank an anonymous reviewer for urging us to make this explicit.

28 For our positive account of how this relevance sensitivity might work see the section ‘Neurodynamics of selective openness’ in Bruineberg and Rietveld (2014).

29 In his recent work, Clark argues that there is no inconsistency between PEM and embodied and enactive approaches towards the mind (Clark 2015a, b). He sees no incompatibility between a theory of the brain as a prediction engine and the openness of the organism to its environment. Clark has argued against a reconstructive account of perception (such as that of Helmholtz) in which the task facing perceptual systems is to form an objective representation of the external world and its causal structure. He argues instead that sensing is inseparable from moving and that the organism acts so as to generate the sensory input it expects, coordinating its behavior with relevant aspects of the distal environment. Clark draws on ML Anderson’s (2014) idea that every perception is accompanied and preceded by some action. Perception can be thought of as opening a sensory channel to the world, and action as controlling the inputs the perceiving animal’s receives via this sensory channel.

30 The recent literature (Clark 2013) on Helmholtzian interpretations of predictive processing contains a number of commentaries from enactivists that overlap with the arguments of this paper in suggestive ways. Froese and Ikegami (2013) criticize Clark (2013) for failing to exclude agents that pursue the “dark room solution”, seeking out a maximally predictable but ecologically unsatisfying environment. Our “crooked scientist argument” (see Sect. 2) can be read as a further extension of this enactive intuition: prediction-error minimization is not the *ultimate aim* for the organism, but rather a *means* by which a better grip is achieved. We suggest that it is only if the generative model embodies the econiche of the organism that minimizing prediction-errors will lead to ecologically relevant outcomes. In another commentary Roesch et al. (2012) criticize Clark for not doing justice to the affective perspective of the organism. The account of the free-energy principle presented in this paper explicitly takes the affective perspective of the organism as its starting point (see p. 16). The solicitation or attractiveness of relevant affordances, which is central in our account, has an affective character.

31 The “primordial soup” simulation in Friston (2013a) consists of the interaction between a number of particles with Newtonian and electrochemical dynamics. When these particles interact with each other over time, the system tends towards a stable configuration in which the system is describable in terms of “internal”, “external”, “perception” and “action” states, based on their causal dependencies. Although not directly causally coupled, the electrochemical dynamics of the internal and external states of the system become synchronized. On Friston’s interpretation, this allows an understanding of the internal dynamics as inferring the hidden dynamics of the environment based on the sensory and action states.

32 We do not mean to argue that coupled clocks are just as much living systems as, for instance, the cell. As mentioned above in footnote 17, additonal conditions are required to demarcate living from non-living systems. Our point here is to highlight the scope of the concept of ‘inference’ that is at play in the free-energy principle. The concept of inference as Friston uses it applies to any process that leads to states with mutual information (Fig. 3).

33 Holding the beam fixed is equivalent to ‘lesioning’ the Markov blanket in Friston (2013a, Figure 5).

34 An important difference between the clocks and the ‘agent’-environment relation is of course that the the agent and environment have different dynamical properties. Although conceptually more clarifying, the phenomenon of synchronization is not limited to systems with identical dynamics, but also applies to coupled non-identical systems (see for instance Pyragas 1997).

35 Importantly, generalized synchrony is not just used to model self-organizing systems. There is a body of literature that relates generalized synchronization to active inference more generally (see for example Friston et al. 2014, 2015 and Friston and Frith 2015).

36 By ‘reflect’, here, we mean that there are internal states of the organism that reliably covary with aspects of the environment, as discussed above in our analysis of the primoridal soup simulation.

37
Anderson and Chemero (2013) raises the important worry that appealing to the concept generative models in a theory of the anticipating brain may lead one down “the lonely and unproductive road” of epistemic internalism (p. 25). We think our interpretation of the generative model avoids this risk because our interpretation is non-representational. We understand the generative model in terms of multiple simultaneous states of action-readiness. These states of action-readiness are selectively sensitive to affordances available in landscape of affordances, which is there independently of any particular individual.

38 Andy Clark raised this objection to us in personal communication and on http://philosophyofbrains.com/2015/12/15/conservative-versus-radical-predictive-processing.aspx.
